# Water diffusion closely reveals neural activity status in rat brain loci affected by anesthesia

**DOI:** 10.1371/journal.pbio.2001494

**Published:** 2017-04-13

**Authors:** Yoshifumi Abe, Tomokazu Tsurugizawa, Denis Le Bihan

**Affiliations:** NeuroSpin, Joliot Institute, Commissariat à l'énergie atomique et aux énergies alternatives, Gif-sur-Yvette, France; University of Oxford, United Kingdom of Great Britain and Northern Ireland

## Abstract

Diffusion functional MRI (DfMRI) reveals neuronal activation even when neurovascular coupling is abolished, contrary to blood oxygenation level—dependent (BOLD) functional MRI (fMRI). Here, we show that the water apparent diffusion coefficient (ADC) derived from DfMRI increased in specific rat brain regions under anesthetic conditions, reflecting the decreased neuronal activity observed with local field potentials (LFPs), especially in regions involved in wakefulness. In contrast, BOLD signals showed nonspecific changes, reflecting systemic effects of the anesthesia on overall brain hemodynamics status. Electrical stimulation of the central medial thalamus nucleus (CM) exhibiting this anesthesia-induced ADC increase led the animals to transiently wake up. Infusion in the CM of furosemide, a specific neuronal swelling blocker, led the ADC to increase further locally, although LFP activity remained unchanged, and increased the current threshold awakening the animals under CM electrical stimulation. Oppositely, induction of cell swelling in the CM through infusion of a hypotonic solution (−80 milliosmole [mOsm] artificial cerebrospinal fluid [aCSF]) led to a local ADC decrease and a lower current threshold to wake up the animals. Strikingly, the local ADC changes produced by blocking or enhancing cell swelling in the CM were also mirrored remotely in areas functionally connected to the CM, such as the cingulate and somatosensory cortex. Together, those results strongly suggest that neuronal swelling is a significant mechanism underlying DfMRI.

## Author summary

It has been reported that neuronal activation results in a decrease of water diffusion in activated neural tissue. This new approach, known as diffusion functional MRI (DfMRI), has high potential for functional imaging of the brain, as the currently widespread blood oxygenation level—dependent (BOLD)-functional MRI (fMRI) method, which is based on neurovascular coupling, remains an indirect marker of neuronal activation. Here, we show that the water apparent diffusion coefficient (ADC) derived from DfMRI increased in specific rat brain regions under anesthetic conditions, reflecting the decreased neuronal activity, especially in regions involved in wakefulness. Electrical stimulation of the central medial (CM) thalamic nucleus exhibiting this anesthesia-induced ADC increase led the animals to transiently wake up. Infusion of the CM with furosemide—a specific blocker of neuronal swelling—led the ADC to increase further locally and increased the current threshold for waking the animals. Conversely, induction of cell swelling in the CM through infusion of a hypotonic solution led to a local ADC decrease and a lower current threshold to wake the animals. Strikingly, the local ADC changes produced by blocking or enhancing cell swelling in the CM were also mirrored remotely in areas functionally connected to the CM, such as the cingulate and somatosensory cortex. Those results strongly suggest that neuronal swelling is a significant mechanism underlying DfMRI.

## Introduction

Diffusion functional MRI (DfMRI) [1,2] has been proposed as an imaging method to investigate brain function noninvasively, an alternative to blood oxygenation level—dependent (BOLD) functional MRI (fMRI) [3], which has been extensively used to investigate brain activity [4]. BOLD-fMRI, which relies on the neurovascular coupling hypothesis and does not directly reflect neuronal activity [5], may fail in some conditions that prevent neurovascular coupling [6]. Besides, BOLD-fMRI is also sensitive to global or local variations in cerebral blood flow and metabolism, which are not necessarily related to underlying neuronal activity, temporally and spatially [7]. Diffusion MRI is thought to be more directly linked to neuronal activation, as the diffusion MRI signal is exquisitely sensitive to minute changes, such as cell swelling, occurring in the tissue microstructure upon various physiological or pathological changes [8]. Indeed, there is a large body of studies reporting that neuronal activation is associated with cell swelling [9–11]. However, while it has been shown that DfMRI signals are not of vascular origin [12], the method has been controversial [13,14], and the exact origin of the DfMRI response remains unclear. To check the hypothesis that the water apparent diffusion coefficient (ADC) measured with DfMRI provides a direct, quantitative assessment of neural states of activity or deactivity and to shed light on the DfMRI mechanisms, we have investigated the functional features of rat brain loci undergoing ADC variations using electrophysiological recordings and electrical stimulation, as well as pharmacological challenges interfering with cell swelling, under various anesthetic or sedative conditions, using several doses of an anesthetic drug, isoflurane, and a sedative drug, medetomidine—two drugs widely used in preclinical MRI studies.

## Results

### DfMRI signals (ADCs) quantitatively reveal network of brain loci affected by anesthesia independently of their vascular status

**Fig 1:**
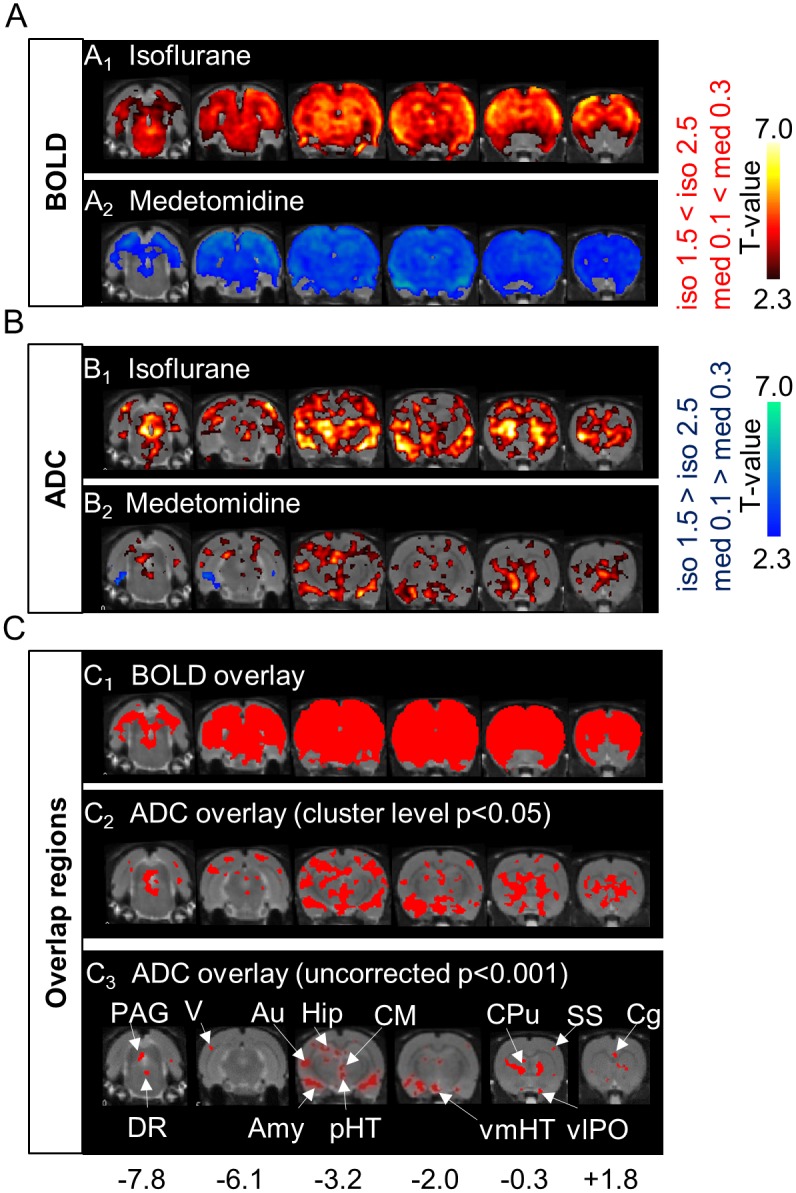
Comparison of Blood Oxygenation Level—Dependent (BOLD) and Apparent Diffusion Coefficient (ADC) maps under both anesthetic conditions. (A, B) T-maps of changes in BOLD signals (A_1_; *n* = 6, A_2_; *n* = 7) and ADC (B_1_; *n* = 10, B_2_; *n* = 8) under isoflurane (iso) and medetomidine (med) dosage conditions (six out of ten slices are shown. Hot colors mean an increase in BOLD or ADC of high dose (cluster-level corrected *p* < 0.05), compared with that of low dose. Cool colors mean the opposite (cluster-level corrected *p* < 0.05). (C_1_) Red regions correspond to regions associated with both a BOLD increase for iso and a BOLD decrease for med (cluster-level corrected *p* < 0.05). (C_2_, C_3_) Red regions correspond to regions showing an ADC increase (C_2_; threshold of cluster-level corrected *p* < 0.05, C_3_; threshold of uncorrected *p* < 0.001) common to both med and iso. With the stricter statistical threshold (C_3_), small regions like the central medial thalamus nucleus (CM) and some hypothalamic nuclei (posterior hypothalamic nucleus [pHT], ventral medial hypothalamic nucleus [vmHT], ventrolateral preoptic nucleus [vlPO]) are easier to see. Numbers at the bottom correspond to the distance (mm) from the bregma. Statistical maps for group analysis and overlay maps of BOLD and ADC can be found in S7 Data.

We first examined the ADC change maps induced by high- and low-dose conditions of both anesthetic agents (isoflurane: 2.5%/1.5% and medetomidine: 0.3 mg/kg/h/0.1 mg/kg/h, see Material and methods). While changes in BOLD signals were widespread in grey and white matter, not specific to any brain location (Fig 1A and 1C_1_), ADC changes were highly localized (Fig 1B), with a significant increase in ADC value (cluster level corrected *p* < 0.05) when increasing anesthetic/sedation agent dosage for both isoflurane and medetomidine (Fig 1B). The BOLD signal, instead, increased with the dose of isoflurane (Fig 1A_1_) but decreased with the increased medetomidine dose (Fig 1A_2_). There were differences, though, in the networks exhibiting this ADC increase between both agents. Under isoflurane, the ADC increase pattern encompassed more regions than with medetomidine, especially the motor cortex, the insular cortex, the posterior thalamic area, and the superior colliculus. The common regions exhibiting the ADC increase under both anesthetic conditions were the cerebral cortex (the somatosensory cortex, the cingulate cortex, the auditory cortex, the visual cortex), the limbic region (the Caudate-Putamen, the hippocampus, the amygdala, the thalamus, the hypothalamus), and the midbrain (the periaqueductal gray, the dorsal raphe [DR]) (Fig 1C_2_ and 1C_3_). Especially, the central medial thalamic nucleus (CM), the posterior hypothalamic nucleus (pHT), the ventral medial hypothalamic nucleus (vmHT), the ventrolateral preoptic nucleus (vlPO), the DR, and the periaqueductal gray (PAG) correspond to a network of regions known to be associated with wakefulness/sleep conditions [15]. As expected, raw diffusion-weighted images that contain a residual T2 (BOLD) contribution to the genuine diffusion component [13,16] showed patterns mixing ADC and BOLD responses, with the diffusion component contributing more to the b1800 maps (S3 Fig). The T2 (BOLD) component was factored out in the ADC maps, by principle.

**Fig 2:**
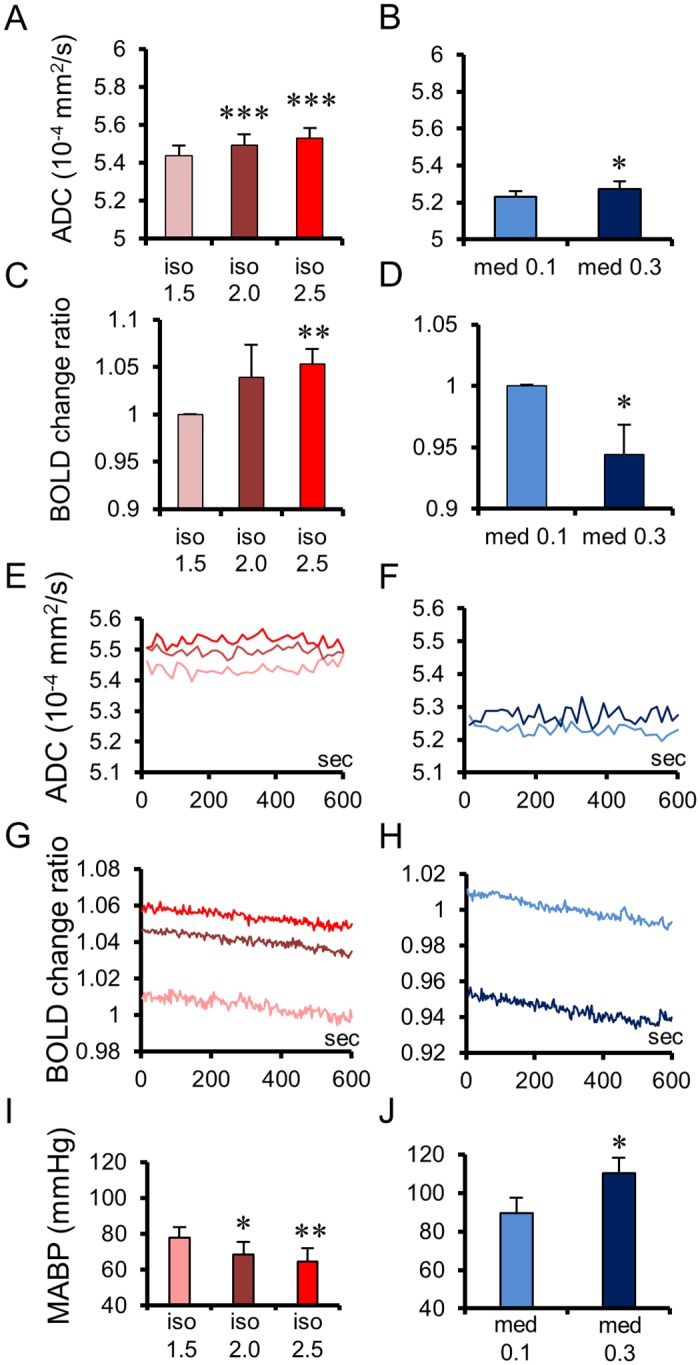
Apparent Diffusion Coefficient (ADC) and Blood Oxygenation Level—Dependent (BOLD) changes and time courses for each dosage of both anesthetic conditions. Averaged ADCs of whole brains for each dosage of isoflurane (A; *n* = 10) and medetomidine (B; *n* = 8). Averaged BOLD signal change ratios of whole brain for each dosage of isoflurane (C; *n* = 6) and medetomidine (D; *n* = 7). Averaged time courses of ADCs (E, F) and BOLD signal changes ratios (G, H) at whole brains for each dosage of isoflurane (E, G) and medetomidine (F, H), showing the stability of the ADC change while the BOLD signal exhibits a significant negative drift with time. Note that the BOLD change levels for each medetomidine dosage are inverted between E and H. Mean arterial blood pressure (MABP) under each dosage of isoflurane (I; *n* = 5) and medetomidine (J; *n* = 5). Bar plots exhibit mean ± standard error of the mean (SEM). * *p* < 0.05, ** *p* < 0.01, *** *p* < 0.001 (Paired *t*-test, versus low dose of each anesthesia). Data for whole brains of individual rats can be found in S1 Data for ADC and S2 Data for BOLD. MABP data of individual rats can be found in S4 Data.

Importantly, the amount of ADC increase was positively correlated with the dose of both anesthetic agents (Fig 2A and 2B and S1 Table). The dose-dependent increases in ADC were significant at 12 brain locations for isoflurane and eight locations for medetomidine (S1 Table) but not at the whole-brain level. On the contrary, the dose-dependent changes of the BOLD signal were significantly correlated to the agent dosage at the whole-brain level, all regions exhibiting the same pattern observed at the whole-brain level (Fig 2C and 2D), but positively for isoflurane and negatively for medetomidine (S1 Table), reflecting (in a reversed way) global MABP (mean arterial blood pressure) changes (Fig 2I and 2J). In addition, the ADC time course was stable over time for all anesthetic conditions (no significant correlation with time) (Fig 2E and 2F), while the average BOLD time-course signal (Fig 2G and 2H) showed a drift, gradually decreasing both for isoflurane and medetomidine, irrespectively of the sign of the dose-dependent relationship (positive for isoflurane and negative for medetomidine). This drift, which is often seen with fMRI experiments and removed by signal detrending, was also observed for the raw diffusion-weighted signals, except under medetomidine 0.3 mg/kg/h, especially at b = 1800s/mm^2^ (S3 Fig), but factored out when calculating the ADC. S2 Table shows the slope values for the ADC and BOLD signal changes with the dosage for each agent. For the ADC, the slope values were the highest in the auditory cortex, the caudate-putamen, and the DR for both agents, indicating that these three regions were more sensitive to those agents. The ADC slopes for both agents at the whole-brain level were smaller than the average slope over the 12 brain locations. The slopes of the BOLD signal in the cerebral cortex and the hippocampus were steeper than that in the limbic regions and the midbrain but with opposite signs for isoflurane and medetomidine. The BOLD slopes for both agents at the whole-brain level were similar to the average slope over the 12 brain locations.

### Increase in water ADC closely mirrors Local Field Potentials (LFPs) patterns, reflecting a decrease in neuronal activity induced by anesthesia

**Fig 3:**
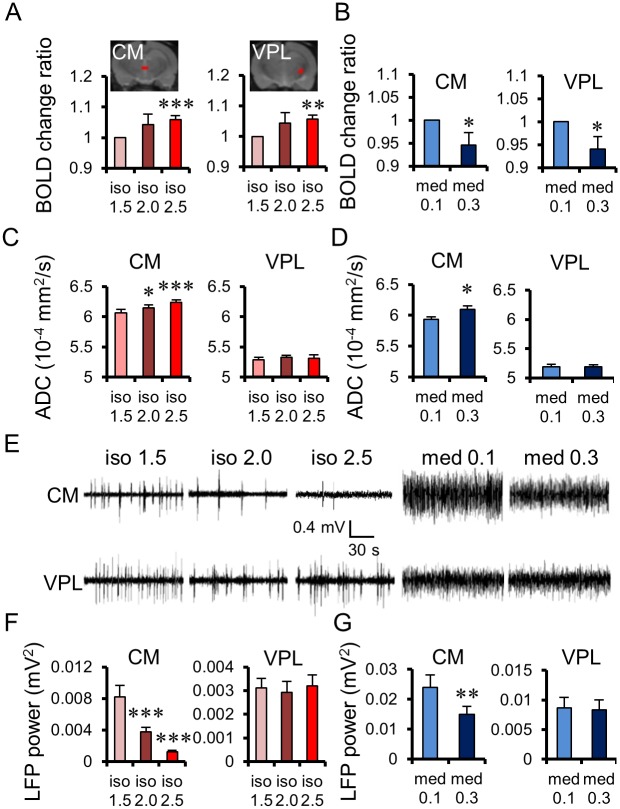
Comparison of Apparent Diffusion Coefficient (ADC) and Blood Oxygenation Level—Dependent (BOLD) changes with Local Field Potentials (LFPs) in the Central Medial (CM) and Ventral Posterolateral (VPL) thalamic nuclei. BOLD changes under each dosage of isoflurane (A) and medetomidine (B) at the CM and the VPL. Region of interest (ROI) locations for the CM and the VPL are overlaid on structural images. ADC changes under each dosage of isoflurane (C) and medetomidine (D) at the CM and the VPL. (E) Representative LFP signals in a single animal at the CM (upper) and the VPL (below), for each dosage of isoflurane and medetomidine. Total LFP power (frequency range: 1–70 Hz) under each dosage of isoflurane (F; *n* = 8 for CM, *n* = 8 for VPL) and medetomidine (G; *n* = 8 for CM, *n* = 8 for VPL). Bar plots exhibit mean ± the standard error of the mean (SEM). * *p* < 0.05, ** *p* < 0.0, *** *p* < 0.001 (Paired *t*-test, versus low dose of each anesthesia). Data for the CM and the VPL of individual rats can be found in S1 Data for ADC and S2 Data for BOLD. LFP power data for the CM and the VPL of individual rats can be found in S4 Data.

To shed light on the relationship between ADC and underlying neuronal activity, we compared the ADC change patterns with recordings of LFPs under the exact same anesthetic conditions (see Materials and methods) in the CM and the ventral posterolateral (VPL) thalamic nuclei. Those regions were chosen because they exhibited different fMRI response patterns: In the CM, the ADC increased with both agent’s dosages, while the BOLD signal increased with isoflurane dosage and decreased with medetomidine dosage (Fig 3A–3D). In contrast, there was no ADC change in the VPL with both anesthetic agents, while there was a positive signal change for BOLD with isoflurane and a negative signal change with medetomidine dosage. From the view point of neural activity, spontaneous firing rates and LFP power clearly decreased in the CM with isoflurane and medetomidine dosage (Fig 3E–3G), while no significant changes in firing rates were observed in the VPL. The decrease in LFP power (see Materials and methods) with agent dosage was highly significant for both agents in the CM, but there were no significant changes in the VPL, although the BOLD signal increased with isoflurane dosage and decreased with medetomidine dosage.

### Electrical stimulation of thalamus CM exhibiting an anesthesia-induced ADC increase leads the anesthetized animals to wake up

**Fig 4:**
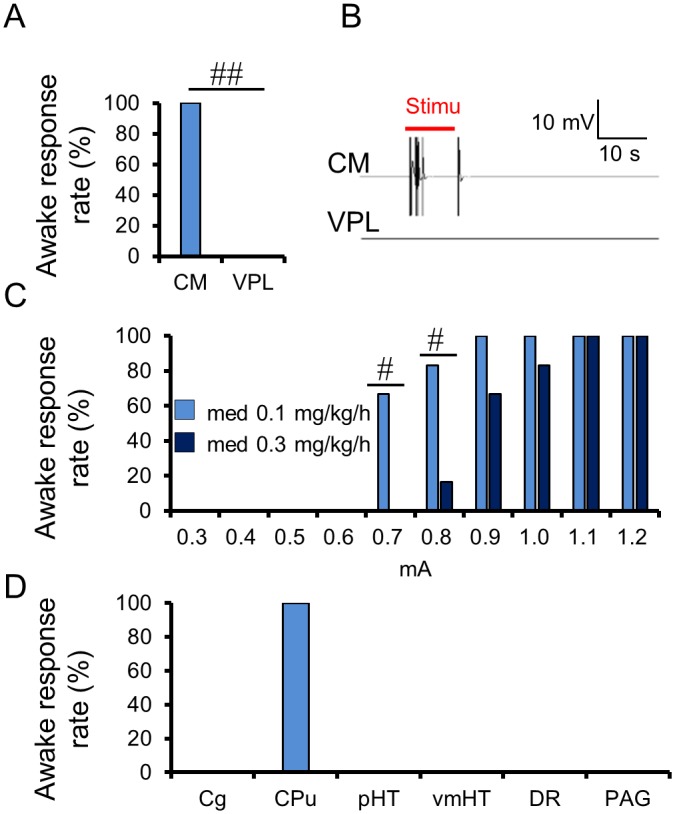
Effects of electrical stimulation of the Central Medial thalamus nucleus (CM) on anesthetic status. (A) Percentage of animals exhibiting an awake response during the electrical stimulation at the CM and VPL under 0.1 mg/kg/h medetomidine condition (*n* = 6). (B) Representative electromyography (EMG) signals of the stimulation, with 0.8 mA amplitude at the CM and the VPL under 0.1 mg/kg/h medetomidine. (C) Percentage of animals exhibiting an awake response during the electrical stimulation at the CM, with an amplitude of 0.3–1.2 mA under 0.1 and 0.3 mg/kg/h medetomidine (*n* = 6). (D) Percentage of animals exhibiting an awake response during the electrical stimulation at the six brain locations under 0.1 mg/kg/h medetomidine (*n* = 4). # *p* < 0.05, ## *p* < 0.01 (Fisher’s exact test). Data for minimum amplitudes of individual rats can be found in S5 Data.

To further confirm the relevance of our findings, an electrical stimulation of the CM and VPL was performed (see Materials and methods). The decrease in LFP power with drug dosage in the CM, in which we observed a dose-dependent ADC increase, reflected a neural deactivation induced by anesthesia or sedation. In contrast, VPL activity seems independent of anesthesia, as no change in ADC nor LFP power could be observed. Indeed, when an electrical stimulation was performed at the CM location under the same medetomidine anesthetic conditions used for the LFP recordings and the MRI experiments the rats exhibited a voluntary movement of their limbs and body, indicating a wakefulness status (Fig 4A and S1 Movie), although they were anesthetized. This animal movement during the stimulation matched the electromyography (EMG) data (Fig 4B). Additionally, the current threshold required to trigger the animal awakening during CM stimulation increased with the dosage of medetomidine (Fig 4C; threshold is 0.75 ± 0.03 mA for medetomidine 0.1 mg/kg/h and 0.93 ± 0.04 mA for medetomidine 0.3 mg/kg/h, *p* < 0.01) and the ADC in the CM (Fig 3D; ADC_CM_ = 5.94 ± 0.03 × 10^−4^ mm^2^/s for medetomidine 0.1 mg/kg/h and ADC_CM_ = 6.09±0.06 ×10^−4^ mm^2^/s for medetomidine 0.3 mg/kg/h, *p* < 0.05) but not the BOLD signal (Fig 3B). Animals returned to an anesthetic status at the end of the stimulation, showing no reflex motor response 1 min after stimulation. In contrast, no wakefulness response could be observed when stimulating the VPL (Fig 4B). In light of the current study, those electrical stimulations and LFP patterns in the CM and the VPL uniquely demonstrate that the ADC signal, indeed, accurately mirrors neural activity changes induced by anesthesia under various anesthetic/sedation conditions, while the BOLD signal appears completely unrelated. Among the other tested loci, only caudate-putamen (CPu) showed a response pattern similar to CM (Fig 4D). There was no wakefulness response in the cingulate cortex (Cg), pHT, vmHT, DR, and PAG, although those regions exhibited an ADC increase upon anesthesia or sedation (Fig 1C_3_). Those results are consistent with earlier results showing the specific roles of the CM and CPu in anesthesia mechanisms [17,18], while the VPL and the other investigated loci do not seem to play a direct role.

### Neuronal swelling inhibition and induction modulate the ADC in the CM and connected loci while changing current threshold to wake up animals under CM electrical stimulation

**Fig 5:**
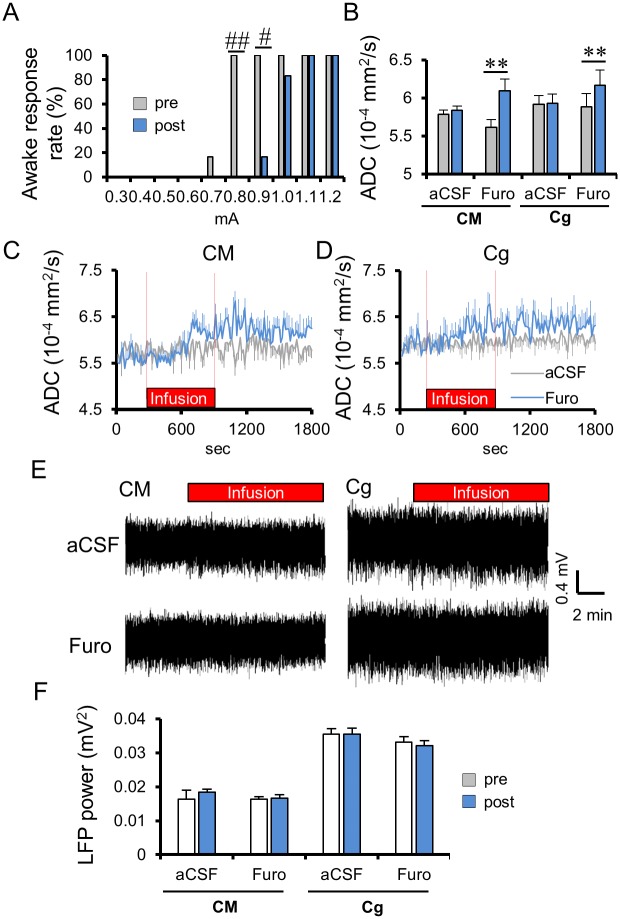
Effects of furosemide infusion in the Central Medial thalamus nucleus (CM) on the Apparent Diffusion Coefficient (ADC), Local Field Potentials (LFPs) and awake response threshold under 0.1 mg/kg/h medetomidine condition. (A) Percentage of animals exhibiting an awake response during CM electrical stimulation after the CM infusion with furosemide (Furo; *n* = 6). (B) Average ADC change in the CM and cingulate cortex (Cg) before and after CM infusion (*n* = 6 for artificial cerebrospinal fluid [aCSF], *n* = 6 for Furosemide). Average time course of ADC change in the CM (C) and Cg (D) with the infusion of aCSF and furosemide. (E) Representative local field potentials (LFP) signals at the CM and Cg with the CM infusion with aCSF (upper) or furosemide (below). (F) Total LFP power (frequency range: 1–70 Hz) in the CM and Cg, before and after CM infusion with furosemide (*n* = 6) or aCSF (*n* = 6). Time course and bar plots exhibit mean ± the standard error of the mean (SEM). ** *p* < 0.01 (two-sided paired *t*-test between pre and post), # *p* < 0.05 (Fisher’s exact test). Data for minimum amplitudes of individual rats can be found in S5 Data. ADC data of individual rats found in S3 Data. LFP power data can be found in S4 Data.

If the DfMRI signal change (and the resulting ADC) is not of vascular origin, the question that next comes to mind is how changes in neuronal activity might result in changes in tissue water diffusion. Diffusion MRI is known to be exquisitely sensitive to minute changes in tissue microstructure [19]. Extra physiological or pathological events resulting in cell swelling are all associated with a decrease in ADC [20–23], but recent studies have shown that such cell swelling could also result in an ADC decrease in more physiological conditions [24]. Hence, the ADC decrease observed during neuronal activation has been tentatively ascribed to a physiological transient cell swelling in the neural tissue, so called neuromechanical coupling hypothesis [2,8]. To check the contribution of cell swelling to the DfMRI signal, we first locally infused in the CM furosemide, an inhibitor of the neuron-specific K^+^/Cl^−^ cotransporter (KCC2), as well as Na^+^/K^+^/Cl^−^ cotransporter (NKCC1) (see Materials and methods), blocking neuronal swelling [25,26] while not inhibiting excitability [26]. Indeed, we did not observe any LFP change in the CM after furosemide or artificial cerebrospinal fluid (aCSF) infusion in CM (Fig 5E and 5F). However, furosemide injection resulted in a significant ADC increase on top of the ADC increase induced by medetomidine, which persisted after the end of the injection (Fig 5B) (as expected, since there was no washout after injection, data were collected for only 15 min, and it would take much longer for furosemide to be metabolized). The ADC increase was more pronounced and significant under the low dose (0.1 mg/kg/h) of medetomidine (from 5.61 ± 0.11 to 6.09 ± 0.15 × 10^−4^ mm^2^/s, *p* < 0.05), while only a not significant trend was visible at the high dose (0.3 mg/kg/h) (from 6.16 ± 0.14 to 6.33 ± 0.12 mm^2^/s, *p* = 0.082) (Fig 5B–5D and S4A and S4B Fig), indicating that residual amounts of dynamic cell swelling were still present at the low dose of medetomidine, but not at the high dose. Indeed, while the LFP power remained unchanged, the possibility of waking up the animals by CM electrical stimulation, which was unchanged under aCSF infusion, required a higher electrical threshold under furosemide infusion (Fig 5A; threshold preinfusion = 0.77 ± 0.02 mA; post infusion = 1.00 ± 0.03 mA, *p* < 0.001), suggesting that blocking cell swelling reinforced the effects of medetomidine. In addition, we found that other brain loci (cingulate cortex and somatosensory cortex) remote from the CM also expressed a further ADC increase during furosemide infusion in the CM (from 5.88 ± 0.17 to 6.16 ± 0.19 × 10^−4^ mm^2^/s in Cg, *p* < 0.01, and from 5.97 ± 0.13 to 6.20 ± 0.15 × 10^−4^ mm^2^/s in the somatosensory cortex [SS], *p* < 0.01) under the low dose (0.1 mg/kg/h) of medetomidine (Fig 5B and S5A and S5C Fig), confirming not only that those areas are functionally connected [18] but also that the residual amount of swelling in the CM neural network left under a low dose of medetomine still has some functional significance remotely. Under a high dose of medetomidine, no further ADC increase could be observed in CM, Cg, and SS (S4 Fig).

**Fig 6:**
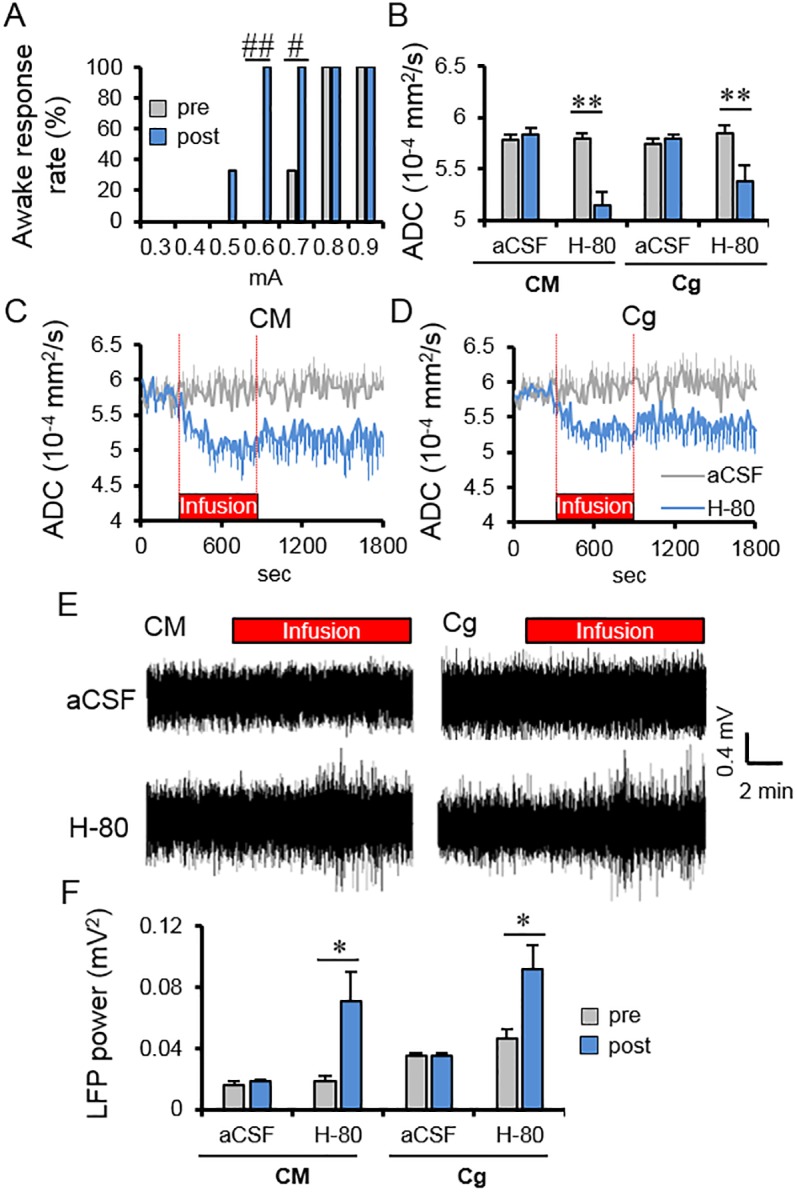
Effects of H-80 infusion in the Central Medial thalamus nucleus (CM) on the Apparent Diffusion Coefficient (ADC), Local Field Potentials (LFPs) and awake response threshold. (A) Percentage of animals exhibiting an awake response during CM electrical stimulation after the CM infusion with −80 milliosmole (mOsm) hypotonic artificial cerebrospinal fluid (aCSF) (H-80; *n* = 6). (B) Average ADC change in the CM and cingulate cortex (Cg) before and after H-80 or aCSF infusion in the CM (*n* = 6 for each condition). Average time course of ADC change at the CM (C) and Cg (D) with the infusion of aCSF and H-80. (E) Representative local field potentials (LFP) signals at CM and Cg during CM infusion with aCSF (upper) or H-80 (low). (F) Total LFP power (frequency range: 1–70 Hz) in CM and Cg before and after CM infusion with H-80 (*n* = 6) or aCSF (*n* = 6). Time course and bar plots exhibit mean ± the standard error of the mean (SEM). ** *p* < 0.01 (Paired *t*-test between pre and post), # *p* < 0.05, ## *p* < 0.01 (Fisher’s exact test). Data for minimum amplitudes of individual rats can be found in S5 Data. ADC data of individual rats found in S3 Data. LFP power data can be found in S4 Data.

Contrary to this, local cell swelling was induced by infusion of a hypotonic solution (−80 milliosmoles [mOsm] aCSF [H-80]) [27,28]. CM infusion with H-80 under a low dose (0.1 mg/kg/h) of medetomidine resulted in a significant ADC decrease not only in the CM (5.79 ± 0.05 to 5.12 ± 0.12 × 10^−4^ mm^2^/s, *p* < 0.01) but also remotely in Cg (5.85 ± 0.07 to 5.38 ± 0.14 × 10^−4^ mm^2^/s, *p* < 0.01) and SS (5.79 ± 0.07 to 5.25 ± 0.12 × 10^−4^ mm^2^/s, *p* < 0.01) (Fig 6B–6D and S5B and S5D Fig). Neuronal activity, through LFP response, was found to increase in CM and Cg after H-80 infusion (Fig 6E). The LFP powers also increased significantly at the CM and the Cg in the post period of infusion (see Materials and methods) (Fig 6F). The current threshold to wake up the animals upon CM electrical stimulation decreased after infusion of H-80 from 0.77 ± 0.02 mA, preinfusion to 0.57 ± 0.02 mA, postinfusion (*p* < 0.001) (Fig 6A).

## Discussion

**Fig 7:**
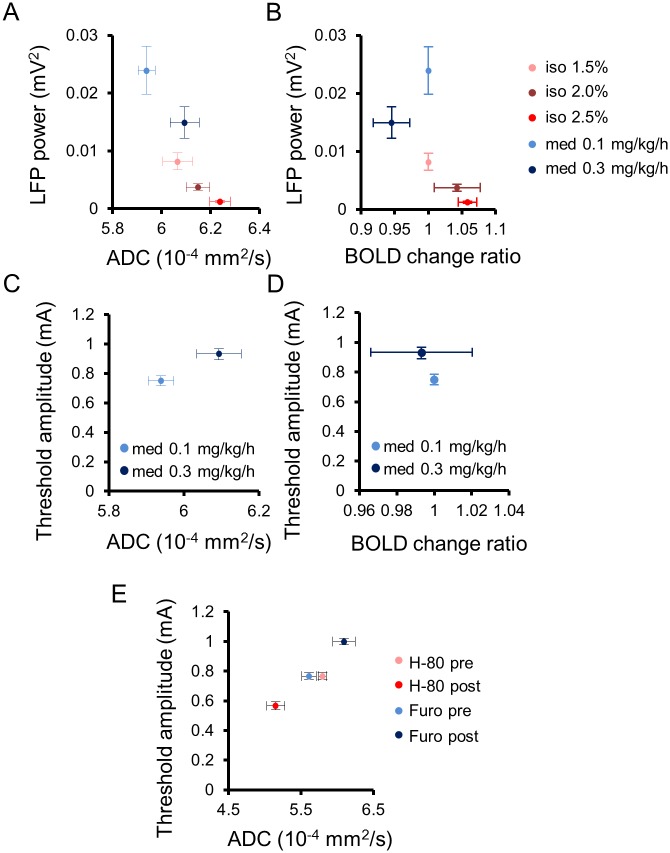
Summarized relationship of the Apparent Diffusion Coefficient (ADC) with underlying neural activity status in the Central Medial thalamic nucleus (CM). Scatter plots between local field potential (LFP) power and ADC (A) or blood oxygenation level—dependent (BOLD) change ratio (B) in the CM under all anesthetic conditions (isoflurane: 1.5%, 2.0%, and 2.5%; medetomidine: 0.1 and 0.3 mg/kg/h). Scatter plots of CM ADC (C) and CM BOLD change ratio (D) with the threshold amplitude to trigger an awake response during CM electrical stimulation under anesthesia with 0.1 and 0.3 mg/kg/h of medetomidine. (E) Scatter plots between CM ADC and the threshold amplitude to trigger an awake response under anesthesia (0.1 mg/kg/h medetomidine) before and after CM infusion of furosemide and H-80. Data with anesthetic dosages for the CM of individual rats can be found in S1 Data for ADC and S2 Data for BOLD. LFP power data with anesthetic dosages for CM of individual rats can be found in S4 Data. Data for minimum amplitudes of individual rats can be found in S5 Data. ADC data for pharmacological diffusion functional MRI (DfMRI) of individual rats found in S3 Data.

DfMRI revealed a dose-dependent, quantitative increase in the water ADC (Fig 1B), independent of the nature of the anesthetic agent, whether isoflurane or medetomidine, reflecting LFP activity in the CM (Figs 3F, 3G and 7A). Following earlier reports that the ADC decreases during neural evoked responses [1,12,29,30], the current findings of a dose-dependent increase of the ADC under both anesthetic drugs indicate a dose-dependent neural deactivation, independent of the nature of the agent. A first important point is that only specific regions exhibited this ADC increase, mainly in the cerebral cortex, the limbic region, and the midbrain, not the whole brain as observed with BOLD fMRI (Fig 1B, 1C_2_ and 1C_3_). The loci exhibiting the ADC increase with isoflurane were more extended than with medetomidine. This difference is consistent with the known enhancement by isoflurane of gamma-aminobutyric acid (GABA)ergic inhibitory systems on many brain areas [31], while medetomidine, which is an α2 adrenergic agonist, enhances neural inhibitory systems [32] through norepinephrine and GABA at selective brain regions [33]. The thalamic area is involved in the wakefulness—sleep cycle [34], and a core of those regions (CM, pHT, vmHT, vlPO, DR, and PAG) corresponds to a network known to be associated with wakefulness—sleep conditions [15,35]. For instance, the preoptic area in the hypothalamus and the tuberomammillary nucleus are involved in the switching and maintaining of a wakefulness state [15]. In the midbrain, the central serotoninergic DR region and the dopaminergic PAG are also responsible for maintaining the arousal condition and the triggering of the wakefulness condition [15]. Especially, the CM has been shown to be involved in the regulation and maintenance of a cortical-thalamic network controlling wakefulness/sedation states. Optogenetical stimulation at high frequency (100 Hz) at the CM of anesthetized rats induce an activation of cerebral cortex (cingulate cortex, medial prefrontal cortex, motor cortex, and somatosensory), striatum, and thalamus, while low frequency (10 Hz) induces a deactivation of the cortex without wakefulness response [18]. Anesthetized rats also show voluntary movement after stimulation of the CM with an inhibitor of the K^+^ channel [17]. A second important point to notice is that the same MRI sequence was used for BOLD and DfMRI. The mismatch between BOLD and ADC changes also further establishes that both methods have different mechanisms and that the ADC change is not of vascular origin, as shown earlier [8]. At very low b values (here we used 10 s/mm^2^) the MRI signal is not sensitized to diffusion but is simply T2-weighted and, thus, only reflects the hemodynamic effect of BOLD. At higher b values, this BOLD contribution remains [13, 16] but does not change with the degree of diffusion weighting (b values). Hence, the ADC calculated from signals acquired with b = 1,000 and 1,800 s/mm^2^ are free of residual T2 (and BOLD) effects, solely reflecting changes in water diffusion in underlying tissues [16].

An advantage of DfMRI is that the derived ADC is an absolute parameter that may directly reflect an increase or a decrease in neuronal activity. To the contrary, an intrinsic limitation of the BOLD signal is that it can only be relative to a baseline or control state and does not have an absolute unit. Although positive responses are common, indirectly associated with neuronal activation, negative responses potentially reflecting deactivation have been elusive [36]. In any case, the BOLD signal, while intrinsically enjoying a higher signal:noise ratio than DfMRI, is more sensitive, by principle, to global or local variations in cerebral blood flow and metabolism, which are not necessarily related to underlying neuronal activity, as exemplified in this study. Although a significant correlation between BOLD signal changes and agent dosages was observed (Fig 2C and 2D and S1 Table), this correlation had opposite signs for two different agents, positive for isoflurane and negative for medetomidine (Fig 7B), at the whole-brain level, following global changes in MAPB (Fig 2I and 2J). The observed BOLD signal decreases, hence, it cannot account for a neuronal deactivation but rather reflects widespread effects of anesthetic agents on the brain vasculature. Indeed, isoflurane is associated with an increase in cerebral blood flow (CBF) due to an overall blood vessel vasodilation [37–43], while medetomidine might induce vasoconstriction [42], perhaps explaining the negative correlation between the BOLD signal (Fig 1A_2_) and MAPB (Fig 2J) we observed by increasing agent dosage.

In this study, the water ADC was found to increase when neural activity decreases by anesthesia conditions, suggesting, by symmetry with DfMRI results obtained upon neuronal stimulation [1], a “shrinking” of cellular elements or, more likely, a return to baseline from an activation state associated with neural cell swelling. Additionally, manipulation of local cell swelling status through very different mechanisms, i.e., the local infusion in the CM of a cell swelling inducer (hypotonic solution, H-80) and a neural swelling blocker (furosemide), established that the ADC very closely reflects local variations in cell size. While we cannot exclude that other mechanisms that variations in cell size might contribute to the DfMRI signal response, we could not find any compatible with both our furosemide and H-80 infusion findings. Such variations in cell size have, in turn, a profound effect on the activity status of the underlying neuronal network, as evidenced from the increase (when blocking neuronal swelling) or decrease (upon cell swelling) of the electrical threshold necessary to wake up the animals by CM electrical stimulation, although they were under anesthesia (Fig 7E). This threshold was also found to be correlated with the absolute ADC value in the CM induced by a different dosage of medetomidine (Fig 7C) but not with the BOLD signal in the CM (Fig 7D). The amount of cell swelling in neuronal networks, of which DfMRI is a biomarker, thus appears closely related to neuronal activity, as further evidenced by the observation that variations of ADCs in the CM were exported remotely in brain regions functionally connected with the CM, such as Cg and SS (Figs 5B and 6B and S5 Fig). The physical mechanisms underlying the ADC decrease upon cell swelling is often thought to be an increase of tortuosity for water diffusion in the reduced extracellular space [44–46]; however, other mechanisms have been proposed, such as the expansion upon the increase of the cell membrane surface of a slow diffusion water molecular layer bound electrostatically to the membranes [23,47,48].

An interesting question is then to elucidate which cell types or elements in the neuropile undergo such functional swelling. Although swelling of astrocytes has been reported [49–53], most studies have shown that swelling is one of the responses associated with neuronal activation [10,54–59], including normal conditions [11,60]. The blocking effect of furosemide on cell swelling is neuron-specific [25,26]. Furosemide is also an antagonist of the α6 subunit of the GABA_A_ receptors [61]. GABA_A_ antagonists (such as bicuculline) usually lead to hyperactivity [26,62] and injection into the CM to wakefulness [63] as well as an ADC decrease [64]. However, this suppression effect of the GABA inhibitory system was not observed in our setup with the furosemide concentration we used. To the contrary, LFP activity remained unchanged, while the ADC and electrical threshold for animal awakening increased, pointing out that LFPs and the ADC are linked to different features of neural tissue function. LFPs are generated by the electrical potentials in multiple neuronal processes, including excitatory, inhibitory interneurons, and astrocytes. Although LFPs integrate “broad” electrophysiological changes, they are not strongly correlated with multi-unit activity (MUA) recordings, which directly reflect electric spiking of neurons. At this stage, one may only speculate that the ADC variations might reflect synaptic activity, originating from the integration at the MRI time scale (much longer than neuronal activity time scale) and spatial resolution of the dynamic swelling and shrinking of a very large number of dendritic spines continuously occurring upon their activation/deactivation status [28,60,65,66], as envisioned already by Ramon y Cajal more than a century ago (“The state of activity would correspond to the swelling and elongation of the [dendritic] spines, and the resting state (sleep or inactivity) to their retraction”) [67]. Dendritic spines are present in a very high density in mammal brain cortical tissue (approximately 1–2 × 10^9^/mm^3^ in the human brain [68], 7 × 10^8^/mm^3^ in the mouse cortex [69]) but not in organotypic slices, which are known to have a drastic reduction in the density of dendritic spines and functional synapses [70], perhaps explaining why neuronal activity did not correlate with any observable ADC change in such slices, although it was observed with cell swelling in extraphysiological conditions [14]. Obviously, further studies will be required to investigate which cell elements or portions might undergo this dynamic swelling process.

In summary, DfMRI (and the derived ADC) appears to provide a faithful assessment of neural activity status, without BOLD confounding hemodynamic effects, the ADC being a suitable biomarker of brain activity in various physiological states or under pharmacological challenges. Contrarily, caution is required when using BOLD fMRI as well as methods built on neurovascular coupling, such as iron-particle based fMRI [71], in conditions in which the brain vascular status is changed, such as during anesthesia, as the BOLD signal sensitivity to peripheral effects on the vasculature might erase smaller changes in hemodynamics induced by a modulation of neuronal activity, a major limitation for preclinical fMRI studies. Overall, this study strongly suggests that neuronal swelling is, indeed, at least one of the mechanisms underlying DfMRI.

## Materials and methods

### Animals

All experiments were performed on 131 male Wister rats (200–300 g, Janvier Labs, Saint Berthevin, France). The rats were housed two per cage under controlled light (7:00–19:00) conditions and were given free access to water and food. All animal procedures in the present study were approved by an Institutional Ethic Committee for Animal Experimentation. The number of animals assigned to each experiment is mentioned in each figure caption.

### Anesthetic time course

The animals were initially anesthetized with 2.5% isoflurane in a gas mixture consisting of air and 20% oxygen for animal preparation. The head of the animal was fixed with ear bars to prevent head motion. For the isoflurane group, the isoflurane concentration was changed to 1.5% during MRI scanner preparation (radiofrequency tuning, magnetic field shimming, and image field-of-view positioning). The first scan initiated after 15 min stabilization time. MR images were then acquired continuously for 10 min under isoflurane dosages of 1.5%, 2.0%, and 2.5%, each time following a 5-min stabilization interval (S1A Fig). For the medetomidine group, the isoflurane anesthesia was discontinued while the animals received a subcutaneous bolus of 0.05 mg/kg medetomidine (Domitor; Pfizer Animal Health, New York, NY, USA) at the end of the animal preparation. An infusion catheter was inserted in a tail vein, allowing a continuous infusion of medetomidine via a syringe pump (Harvard Apparatus; Holliston, MA, USA). Isoflurane was switched off at the start of the medetomidine injection. MR images were acquired continuously for 10 min under medetomidine dosages of 0.1 and 0.3 mg/kg/h, each time following a 30-min stabilization interval, by reference to a previous report [72].

### MRI acquisitions

For all MRI experiments, the animals were intubated and mechanically ventilated (MRI ventilator; CWE Inc., Ardmore, PA, USA). The respiration rate (50 bpm), end-tidal CO_2_ (4%–5%), and rectal temperature (37°C) of the animals were monitored and artificially controlled in the normal range during scanning. The body temperature was maintained at 37°C using an MR-compatible, feedback-controlled air heating system (model 1025; SA Instruments, Stony Brook, NY, USA). MABP was measured from the tail artery outside the magnet. MRI experiments were performed using a horizontal bore, 7T MRI scanner (PharmaScan; Bruker, Ettlingen, Germany) equipped with a gradient system allowing a maximum gradient strength of 760 mT/m. A quadrature volume coil (inner diameter is 38 mm; Bruker, Ettlingen, Germany) was used for transmission and reception. First, we determined the position of ten slices of the brain between +5.0 and −12.0 mm from the bregma, using anatomical images acquired along three orthogonal directions. We acquired DfMRI images using a diffusion-sensitive double spin echo (SE) echo planar imaging (EPI) sequence with following parameters; TR = 2,500 ms, TE = 32 ms, FOV = 2.5 × 2.5 cm^2^, Matrix = 100 × 100, Slices number = 10, Slice thickness = 1.5 mm, Slice gap = 0.2 mm, Resolution = 0.25 × 0.25 × 1.5 mm^3^, Acquisition bandwidth = 357 kHz, 1 average and 1 shot, b-values = 1,000 and 1,800 mm^2^/s along three directions; {X = 1, Y = 0, Z = 0}, {0, 1, 0}, and {0, 0, 1}. BOLD images were acquired with the same parameters with TR = 3,000 ms and a b-value of 10 mm^2^/s along {1, 1, 1}. Scan time for DfMRI and BOLD images was 10 min. Forty volumes were acquired for DfMRI and 200 for BOLD, resulting in time resolutions of 15 s and 3 s for DfMRI and BOLD, respectively. After the acquisition of DfMRI or BOLD images, structural (anatomical) images were obtained using a multislice Rapid Acquisition with Relaxation Enhancement (RARE) sequence at the same brain locations with the following parameters; TR = 2,500 ms, effective TE = 60 ms, FOV = 2.5 × 2.5 cm^2^, Matrix = 256 × 256, Slices number = 10, Slice thickness = 1.5 mm, slice gap = 0.2 mmm, Resolution = 0.097 × 0.097 × 1.5 mm^3^, Average = 10.

### MRI data analysis

SPM8 software (Welcome Trust Centre for Neuroimaging, London, UK) and in-house software written in Matlab [12] were used for data preprocessing and statistical analysis. Image preprocessing was performed first individually for each animal. Time-series images (DfMRI images with b1000 and b1800, and BOLD images) for each drug dosage were realigned to correct for residual head motion and coregistered to the reference structural images. Then, all images where spatially normalized and coregistered to the Paxinos and Watson rat brain atlases [73]. Finally, these images were resliced to a resolution of 0.2 × 0.2 × 0.2 mm^3^ and smoothed with a full width at half maximum (FWHM) Gaussian kernel of 0.6 mm.

Time-series ADC maps were calculated separately for each animal at each drug dosage as ADC = ln(S_b1000_/S_b1800_)/800, in which S_b1000_ and S_b1800_ are the signal acquired with b = 1000 and 1,800 s/mm^2^, using the preprocessed time-series DfMRI images to remove any residual T2/T2* (BOLD) effects [1], and averaged over the three directions to increase signal noise ratio and remove residual differences in gradient hardware (diffusion anisotropy effects were ignored in the regions of interest (ROIs) used for analysis at our spatial resolution). ADCs calculated with high b values (also called “synthetic ADC” or “shifted ADC” [sADC]), instead of typical values of 0 and 1,000 s/mm^2^, have more emphasis toward nonGaussian diffusion and water interaction with cell membranes [74]. Time-series BOLD signal maps for each animal at each drug dosage were normalized by the average value of the BOLD signal at a whole brain for each low dosage, respectively, as the BOLD signal is relative. Time-series ADC maps, which correspond to absolute measurements, were not normalized. The time-series maps of the absolute ADC values and normalized BOLD signals for each animal at each drug dosage were finally pooled for a group analysis. Then, a two-way repeated ANOVA analysis (cluster level corrected *p* < 0.05) was performed over the whole time courses and dosage conditions for isoflurane and medetomidine, respectively. Statistical ADC and BOLD maps were overlaid on the brain structural image. Overlay maps were created using MRIcron (http://www.cabiatl.com/mricro/mricro/mricro.html) to show regions commonly affected by isoflurane and medetomidine, for DfMRI and BOLD, respectively.

ADC and BOLD time-course data were extracted for each drug dosage and for each animal from the clusters visible on the ADC overlay maps of Fig 1C_3_ (uncorrected *p* < 0.001), using MarsBar (MRC Cognition and Brain Sciences Unit, Cambridge, UK). Data were first averaged at each time point over all voxels of each ROI on an individual animal basis, then averaged for each ROI over all animals. The ROI number of voxels were 171 for SS, 58 for M, 79 for V, 180 for Au, 24 for Cg, 313 for CPu, 92 for Hip, 738 for Amy, 95 for Tha, 66 for HT, 58 for DR, and 112 for PAG, respectively. Abbreviations of the brain regions that were used in this report are shown in S3 Table. ROIs corresponding to CM (21 voxels) and VPL (19 voxels) were manually drawn using MRIcron (Fig 3A). Time-series S_b1800_ and S_b1000_ signal maps after averaging over the three directions for each animal at each drug dosage were normalized by the average value of the S_b1800_ and S_b1000_ at a whole brain for each low drug dosage, respectively. Then, statistical S_b1800_ and S_b1000_ maps were also produced by two-way repeated ANOVA analysis (cluster level corrected *p* < 0.05) over the whole time courses and dosage conditions for isoflurane and medetomidine, respectively. Averaged S_b1800_ and S_b1000_ of each anesthetic dosage were calculated in the same way as the BOLD signal using the same ROIs.

To provide some indication of the overall signal level in our image data, signal:noise ratios (SNRs) were estimated at whole brain for each animal and for each of the first five raw time-course images (before preprocessing and for each direction separately for the DfMRI data) under 0.1 mg/kg/h medetomidine. Averaged SNR values (mean ± SEM) were 8.51±0.25 for b1000 (*n* = 8), 5.57 ± 0.17 for b1800 (*n* = 8), and 11.32 ± 0.69 (*n* = 7) for BOLD.

### LFP recording and analysis

LFP recording was performed outside the MRI scanner. The animals were initially anesthetized with 1.5%–2.0% isoflurane to insert a recording electrode under stereotaxic condition. The tungsten micro wire monopolar electrode (1.0 MΩ, 3-μm tip and 125-μm shaft diameter; MicroProbe, MD, USA) was inserted into the CM (−0.4 mm ML, −3.7 mm AP, −6.5 mm DV from the bregma), the VPL (−3.3 mm ML, −3.0 mm AP, −6.5 mm DV), and the Cg (−0.5 mm ML, +1.8 mm AP, −3.0 mm DV). The electrode was connected to a differential AC amplifier Model 1700 (AM systems, Sequim, WA, USA) via a Model 1700 head stage (AM systems, Sequim, WA, USA). The reference electrode (set to ground) was inserted into the scalp. LFP data was recorded using a Powerlab 8/35 with LabChart (AD Instruments; Dunedin, New Zealand) with a bandpass of 1–500 Hz and digitized at 1.0 kHz. The line power 50 Hz noise was eliminated from the signal using selective filters. During the LFP recording, the rat’s rectal temperature was maintained at 37°C using a heating pad (DC temperature controller; FHC Inc., Bowdoin, ME, USA). CO_2_ concertation, respiration rate, and MABP were not monitored and controlled during LFP recording. The LFP recording scheme of Fig 3E under the various anesthetic conditions was identical to that used for the MRI acquisitions (Fig 1A); however, for each 10 min of LFP recordings, the initial 2.5 min and the last 2.5 min were discarded, keeping only the central 5 min for analysis with an in-house MATLAB program. Total LFP power (mean square voltage) was calculated within a frequency range of 1–70 Hz, covering delta to gamma frequency bands.

### Pharmacological DfMRI

A local injection of −80 mOsm hypotonic aCSF (H-80) for inducing cell swelling [27,28] and furosemide (Sigma-Aldrich, St. Louis, MO, USA) for blocking neural swelling [25,26] into the CM was performed to investigate effects on ADC and neural status. A cannula (8-μm diameter; PlasticsOne, Roanoke, VA, USA) was inserted into the CM under stereotaxic conditions and anesthesia with 1.8%–2.0% isoflurane. The cannula was fixed with a dental cement (GC Unifast Trad; GC CO., Tokyo, Japan) and an adhesive (Super-Bond C and B; Sun Medical CO., LTD., Shiga, Japan). DfMRI experiments were performed 1 day after surgery using the paradigm described above under 0.1 and 0.3 mg/kg/h medetomidine but using a homemade surface RF coil (a single loop diameter is 2.7 cm) for transmission and reception, as the cannula set-up was not compatible with the volume coil. Before acquisition of images, the RF excitation power was adjusted to the CM and the Cg. The DfMRI data were acquired with the same parameters as the resting state DfMRI experiment with several dosages of isoflurane and medetomidine for 30 min (120 volume) total. The animals were intubated and mechanically ventilated. The respiration rate (50 bpm), end-tidal CO_2_ (4%–5%), and rectal temperature (37°C) of the animals were monitored and artificially controlled in the normal range during scanning. But MABP was not monitored. A 10-min infusion with normal osmolality aCSF (299.95 mOsm; in mM: 140 NaCl, 3.0 KCl, 1.0 MgCl_2_, 0.3 NaH_2_PO_4_, 1.2 Na_2_HPO_4_, 3.0 glucose, 1.25 CaCl_2_, Sigma-Aldrich, St. Louis, MO, USA), H-80 (219.95 mOsm) or 1.5 mM furosemide using a 10-μl Hamilton syringe and a syringe pump (Kent Scientific Corporation, Torrington, CT, USA) was performed 5 min after the start of the DfMRI acquisition (S1B Fig). After finishing the infusion, additional DfMRI data were acquired for 15 min without washing out the infused solution. These solutions were infused at a flow rate of 0.1 μl/min for 10 min (1.0 μl total). RARE images were also acquired at the end of the DfMRI acquisition to verify the cannula position in the CM (S1D Fig). H-80 was made by adding distilled water on the normal osmolality aCSF (27). The ADCs at the CM, the Cg, and the SS were calculated by averaging at 5-min pre- and postperiods (S1B Fig). ADC time courses were calculated in the same way as the experiment of DfMRI with different pharmacological conditions. ROIs corresponding to the CM (15 voxels), the Cg (49 voxels), and the right SS (68 voxels) were manually drawn using MRIcron (S1E and S5E Figs).

SNRs of pharmacological DfMRI images (b1000 and b1800) were individually calculated in the same way as the experiment of DfMRI with different drug dosages, using the first five images, which were obtained before aCSF infusion under 0.1 mg/kg/h medetomidine condition. The SNRs (mean ± SEM) are 12.15 ± 0.48 for b1000 (*n* = 6) and 8.41 ± 0.33 for b1800 (*n* = 6).

### Electrical stimulation

Electrical stimulation experiments were performed outside the MRI scanner. The animals were initially anesthetized with 1.5%–2.0% isoflurane to insert electrodes (bipolar tungsten wire electrodes, 200-μm tip and 230-μm shaft diameter; PlasticsOne, Roanoke, VA, USA) under stereotaxic conditions. For the stimulation, isoflurane was replaced by medetomodine at a dosage of 0.1 and 0.3 mg/kg/h. This experiment was performed only under the medetomidine condition because the ADC increase for medetomidine was more specific than that for isoflurane (See Fig 1B). During the experiment, the rat’s rectal temperature was maintained at 37°C using the heating pad. Electrical stimulation at 100 Hz was performed with a pulse width of 0.4 ms, with an intensity of 0.8 mA, for 10 s using an isolated pulse stimulator (Model 2100, AM systems, Sequim, WA, USA). The electrical stimulation paradigm was applied in six rats in the CM and VPL and further applied on four animals in six other locations, based on observed fMRI responses: Cg (−0.5 mm ML, +1.8 mm AP, −3.0 mm DV), CPu (−2.4 mm ML, +0.35 mm AP, −5.0 mm DV), pHT (−0.4 mm ML, −3.7 mm AP, −8.3 mm DV), vmHT (−0.4 mm ML, −2.1 mm AP, −9.4 mm DV), DR (0 mm ML, −7.9 mm AP, −6.2 mm DV), and PAG (−0.6 mm ML, −7.9 mm AP, −5.8 mm DV) under 0.1 mg/kg/h medetomidine, corresponding to Fig 4D. A needle electrode was also inserted to the right quadriceps femoris muscle with a reference electrode inserted into the skin of the right limb to record EMG activity. EMG signals were recorded using a Powerlab 8/35 with LabChart (AD Instruments, Dunedin, New Zealand), as well as animal behavior using a video camera. The recording was continuously made over 1 min, starting 10 s before electrical stimulation after the recording of each stimulation. A reflex test of the rat’s limbs was performed to check the animal anesthesia status. EMG data were analyzed using an in-house MATLAB program.

In the stimulation experiment corresponding to Fig 4C, electrical stimulations at 100 Hz for each medetomidine dosages were performed at the CM with a pulse width of 0.4 ms, with an increasing intensity of 0.3–1.2 mA, for 10 s. The interval time between each intensity of the electrical stimulation was 3 min (S2A Fig). Minimum amplitude (mA) with which the anesthetized exhibited a wakefulness response during CM stimulation was defined as the threshold amplitude to wake up. Electrical stimulations corresponding to Figs 5A and 6A were performed at the CM with the same parameters as S2A Fig before and after a 10 min-infusion of H-80 and furosemide into the CM under the 0.1 mg/kg/h medetomidine condition (S2B Fig). Before the stimulation, a bipolar electrode (tungsten wire electrodes, 200-μm tip and 230-μm shaft diameter) fused with a cannula (8-μm diameter; PlasticsOne, Roanoke, VA, USA) and a tungsten micro wire monopolar electrode (1.0 MΩ, 3-μm tip and 125-μm shaft diameter; MicroProbe, MD, USA) were inserted into the CM, and the tungsten micro wire monopolar electrode was inserted into the Cg of initially anesthetized animals with 1.5%–2.0% isoflurane. LFP recording at the CM and the Cg was performed 5 min after the start of the 10-min infusion (S2B Fig). Total LFP power (mean square voltage) at the CM and the Cg were calculated within a frequency range of 1–70 Hz at 5-min pre- and postperiods (S2B Fig).

## Supporting information

S1 Fig Time courses of DfMRI and pharmacological DfMRI studies. (A) Experimental time course of anesthetic dosages of isoflurane (iso) and medetomidine (med). (B) Time course corresponding to Figs 5B–5D and 6B–6D for DfMRI, and S4 Fig with CM infusion of furosemide, H-80 or aCSF under 0.1 mg/kg/h medetomidine condition. The insertion the cannula was performed one day before the MRI scan. The 10 min-infusion was performed 5 min after the start of the DfMRI acquisition. Average ADCs were calculated at the pre- and post-periods of the infusion. (C) Representative raw images of BOLD and DfMRI (b1000 and b1800) acquired with the volume RF coil under 0.1 mg/kg/h medetomidine condition. (D) Structural (RARE) image showing the position of the inserted cannula. (E) Representative raw images of DfMRI (b1000 and b1800) at CM and Cg slices acquired with the surface RF coil under 0.1 mg/kg/h medetomidine condition. Red regions show the ROIs of the CM and Cg.
(TIF)

S2 Fig Time courses of electrical stimulation studies. (A) Time course corresponding to Fig 4C. The insertion of an electrode was conducted under isoflurane. The electrical stimulations (red arrows) with an amplitude of 0.3–1.2 mA at the CM were performed under 0.1 and 0.3 mg/kg/h medetomidine dosages. Resting time was 3 min for each stimulation and 30 min for each dosage. For each stimulation period, an EMG and a video recordings were performed over 1 minute, starting 10 seconds before electrical stimulation. (B) Time course corresponding to Figs 5A and 6A for the CM infusion with furosemide or H-80 and the electrical stimulations under 0.1 mg/kg/h medetomidine condition. Electrical stimulations (red arrows) was performed before and after the 10 min-infusion. The infusion was performed 5 min after the start of LFP recording. LFP powers were calculated at the pre- and post-periods of the infusion.
(TIF)

S3 Fig S_b1800_ and S_b1000_ changes for each dosage of both anesthetic conditions. T-maps of changes in S_b1800_ signals (A; n = 10 for iso and n = 8 for med) and S_b1000_ signals (B; n = 10 for iso and n = 8 for med) under isoflurane and medetomidine dosage conditions (6 out of 10 slices are shown). Hot colors mean an increase in S_b1800_ and S_b1000_ signals of high dose (cluster level corrected p&lt;0.05), compared with that of low dose of each anesthesia. Cool colors mean the opposite (cluster level corrected p<0.05). The number of the below shows the distance (mm) from the bregma. Averaged S_b1800_ (C, E) and S_b1000_ (D, F) signals change ratios at whole brains for each dosage of isoflurane (C, D) and medetomidine (E, F). Time course of S_b1800_ (G, I) and S_b1000_ (H, J) signals change ratios at whole brains for each dosage of isoflurane (G, H) and medetomidine (I, J). Bar plots exhibit mean ± s.e.m. Data for S_b1800_ and S_b1000_ in whole brains of individual rats can be found in S6 Data. Statistical maps for group analysis of S_b1800_ and S_b1000_ can be found in S7 Data.
(TIF)

S4 Fig Effects of furosemide CM infusion under 0.3 mg/kg/h medetomidine condition. (A) Average time course of ADC change at CM with the infusions of furosemide and aCSF. Average ADC changes in the CM (B), the Cg (D), and the SS (E) pre- and post-infusion of furosemide (n = 6 for each dosage) or aCSF (n = 6 for med 0.1 and n = 5 med 0.3) under 0.1 and 0.3 mg/kg/h medetomidine dosages. (C) Total LFP power (frequency range: 1–70 Hz) at the CM pre- and post-CM infusion with furosemide (n = 6 for each dosage) or aCSF (n = 6 for each dosage) under 0.1 and 0.3 mg/kg/h medetomidine dosages. Time course and bar plots exhibit mean ± s.e.m. * p<0.05, ** p<0.01 (Paired t-test between pre and post). ADC data of individual rats found in S3 Data. LFP power data of individual rats can be found in S4 Data.
(TIF)

S5 Fig Effects of CM infusion with furosemide, H-80, or aCSF in the right somatosensory cortex. Average time course of ADC change at the SS with CM infusion of aCSF (n = 6), furosemide (A; n = 6), or H-80 (B; n = 6) (SS) under 0.1 mg/kg/h medetomidine condition. The average ADC at the SS pre- and post-CM infusion of aCSF, furosemide (C), or H-80 (D). (E) ROI location (red region) in SS overlaid on the representative DfMRI image of b1000. Time course and bar plots exhibit mean ± s.e.m. ** p<0.01 (Paired t-test between pre and post). ADC data of individual rats found in S3 Data.
(TIF)

S1 Table Dose-dependent change of ADC and BOLD under both anesthetic drugs. Correlation of absolute ADC value or the ratio of BOLD signal change with anesthetic doses at 12 locations and in the whole brain (r: correlation coefficient, p: p value). * p<0.05, ** p<0.01, *** p<0.001. Data for 12 brain locations and whole brain of individual rats can be found in S1 Data for ADC and S2 Data for BOLD.
(DOCX)

S2 Table Slopes of ADC and BOLD changes with agent dosage for each anesthetic drug. The slope was estimated by dividing the absolute ADC changes or the relative BOLD signal change with anesthetic agent dose changes. * Regions with a significant correlation (p<0.05) as per S1 Table. A.U. = arbitrary units. Data for 12 brain locations and whole brain of individual rats can be found in S1 Data for ADC and S2 Data for BOLD.
(DOCX)

S3 Table Abbreviations of brain regions. (DOCX)

S1 Data ADC data of DfMRI. Time-course data of ADCs at all brain locations and a whole brain with isoflurane and medetomidine dosages for Figs 2, 3 and 7, S1 and S2 Tables.
(XLSX)

S2 Data BOLD data. Time-course data of BOLD signals at all brain locations and a whole brain with isoflurane and medetomidine dosages for Figs 2, 3 and 7, S1 and S2 Tables.
(XLSX)

S3 Data ADC data of pharmacological DfMRI. Time-course data of ADCs at CM, Cg, and SS with infusion of furosemide, H-80, and aCSF for Figs 5 and 6, S4 and S5 Figs.
(XLSX)

S4 Data MABP and LFP data. MABP data for Fig 2I and 2J. LFP power with isoflurane and medetomidine dosages for Figs 3 and 7. LFP power before and after infusion of furosemide, H-80, and aCSF for Figs 5, 6 and 7 and S4 Fig.
(XLSX)

S5 Data Values of minimum amplitude of an awake response. Minimum amplitudes exhibiting an awake response for Figs 4C, 5A, 6A and 7.
(XLSX)

S6 Data S_b1000_ and S_b1800_ data of DfMRI. Time-course data of S_b1000_ and S_b1800_ at a whole brain with isoflurane and medetomidine dosages for S3 Fig.
(XLSX)

S7 Data Statistical and overlay maps. SPM statistical maps for group analysis of BOLD for Fig 1A, ADC for Fig 1B, S_b1800_ for S3A Fig, and S_b1000_ for S3B Fig. Overlay maps of BOLD and ADC for Fig 1C, representatively. These maps are in analyze format and can be opened with freely available image viewers such as MRIcron.
(7Z) Click here for additional data file.

S1 Movie Typical awake response of an anesthetized rat during CM electrical stimulation. The rat which was anesthetized with 0.1 mg/kg/h medetimidine received an electrical stimulation with an amplitude of 0.8 mA at the CM for 10s. During the stimulation, the anesthetized rat exhibited a voluntary movement of limbs and body. Movements disappeared at the end of the stimulation.
(MOV)

## Abbreviations

aCSF: artificial cerebrospinal fluid
ADC: apparent diffusion coefficient
BOLD: blood oxygenation level—dependent
CBF: cerebral blood flow
Cg: cingulate cortex
CM: central medial thalamus nucleus
CPu: caudate-putamen
DfMRI: diffusion functional MRI
DR: dorsal raphe
EMG: electromyography
fMRI: functional MRI
GABA: gamma-aminobutyric acid
iso: isoflurane
LFP: local field potential
MABP: mean arterial blood pressure
med: medetomidine
mOsm: milliosmole
MUA: multi-unit activity
PAG: periaqueductal gray
pHT: posterior hypothalamic nucleus
ROI: region of interest
SEM: standard error of the mean
SS: somatosensory cortex
vlPO: ventrolateral preoptic nucleus
vmHT: ventral medial hypothalamic nucleus
VPL: ventral posterolateral thalamus nucleus
