# Supplementary figures and images for: Water diffusion closely reveals neural activity status in rat brain loci affected by anesthesia
Source: PLoS Biol. 2017 Apr 13;15(4):e2001494. doi: 10.1371/journal.pbio.2001494 (PMC5390968; doi:10.1371/journal.pbio.2001494)

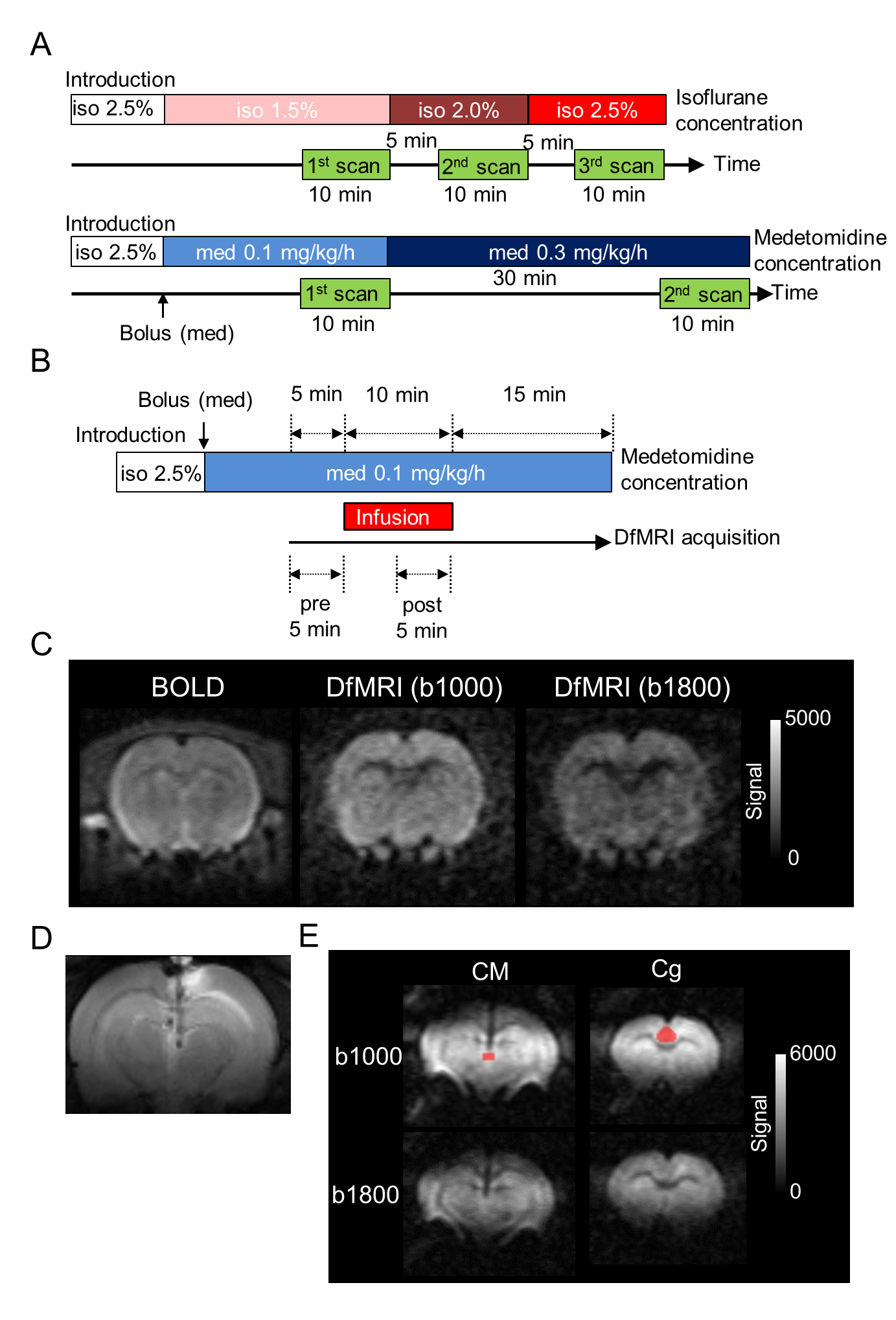

Supplement: S1 Fig — (A) Experimental time course of anesthetic dosages of isoflurane (iso) and medetomidine (med). (B) Time course corresponding to Figs 5B–5D and 6B–6D for DfMRI, and S4 Fig with CM infusion of furosemide, H-80 or aCSF under 0.1 mg/kg/h medetomidine condition. The insertion the cannula was performed one day before the MRI scan. The 10 min-infusion was performed 5 min after the start of the DfMRI acquisition. Average ADCs were calculated at the pre- and post-periods of the infusion. (C) Representative raw images of BOLD and DfMRI (b1000 and b1800) acquired with the volume RF coil under 0.1 mg/kg/h medetomidine condition. (D) Structural (RARE) image showing the position of the inserted cannula. (E) Representative raw images of DfMRI (b1000 and b1800) at CM and Cg slices acquired with the surface RF coil under 0.1 mg/kg/h medetomidine condition. Red regions show the ROIs of the CM and Cg. (TIF) [file pbio.2001494.s001.TIF]

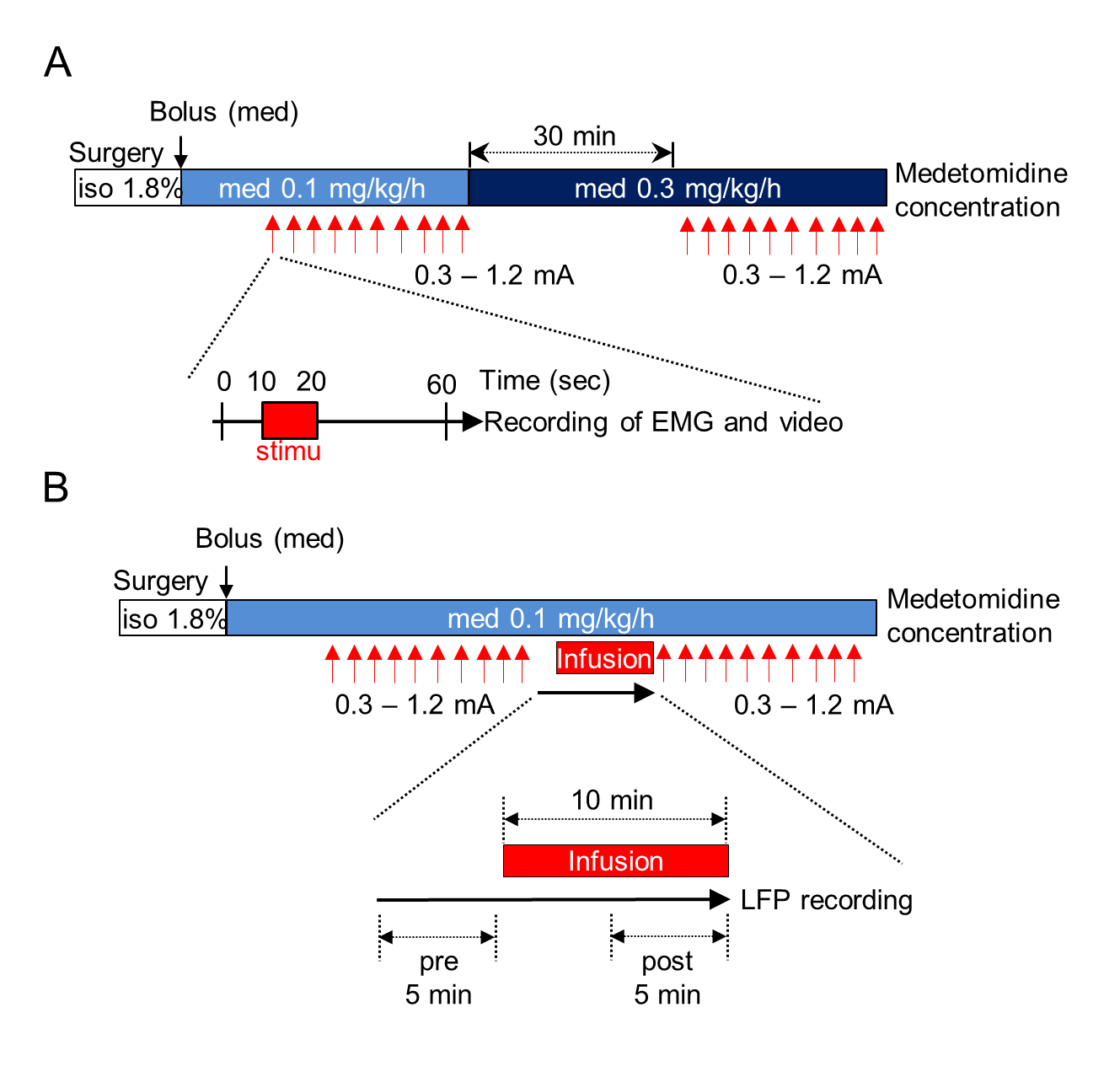

Supplement: S2 Fig — (A) Time course corresponding to Fig 4C. The insertion of an electrode was conducted under isoflurane. The electrical stimulations (red arrows) with an amplitude of 0.3–1.2 mA at the CM were performed under 0.1 and 0.3 mg/kg/h medetomidine dosages. Resting time was 3 min for each stimulation and 30 min for each dosage. For each stimulation period, an EMG and a video recordings were performed over 1 minute, starting 10 seconds before electrical stimulation. (B) Time course corresponding to Figs 5A and 6A for the CM infusion with furosemide or H-80 and the electrical stimulations under 0.1 mg/kg/h medetomidine condition. Electrical stimulations (red arrows) was performed before and after the 10 min-infusion. The infusion was performed 5 min after the start of LFP recording. LFP powers were calculated at the pre- and post-periods of the infusion. (TIF) [file pbio.2001494.s002.TIF]

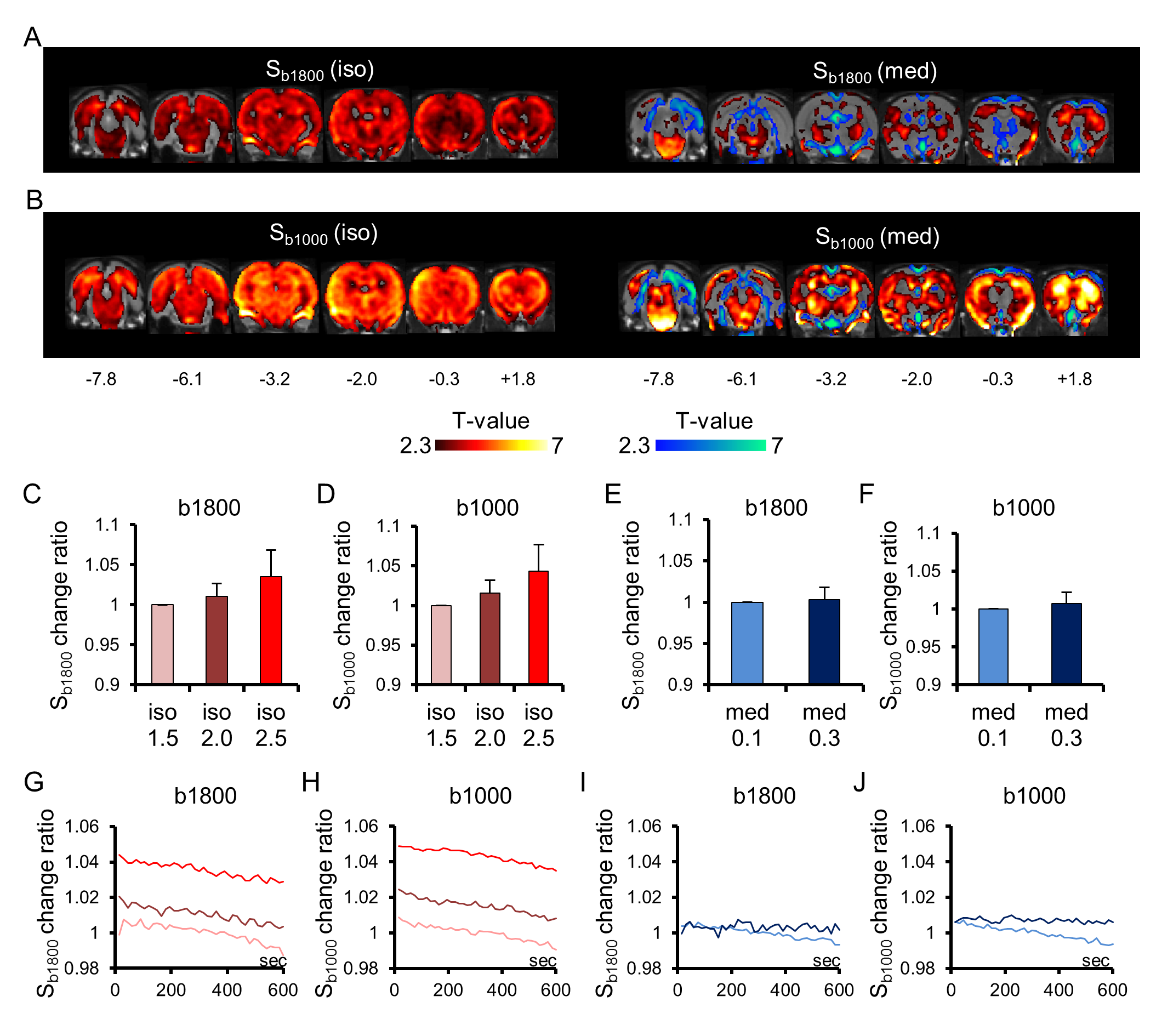

Supplement: S3 Fig — T-maps of changes in Sb1800 signals (A; n = 10 for iso and n = 8 for med) and Sb1000 signals (B; n = 10 for iso and n = 8 for med) under isoflurane and medetomidine dosage conditions (6 out of 10 slices are shown). Hot colors mean an increase in Sb1800 and Sb1000 signals of high dose (cluster level corrected p<0.05), compared with that of low dose of each anesthesia. Cool colors mean the opposite (cluster level corrected p<0.05). The number of the below shows the distance (mm) from the bregma. Averaged Sb1800 (C, E) and Sb1000 (D, F) signals change ratios at whole brains for each dosage of isoflurane (C, D) and medetomidine (E, F). Time course of Sb1800 (G, I) and Sb1000 (H, J) signals change ratios at whole brains for each dosage of isoflurane (G, H) and medetomidine (I, J). Bar plots exhibit mean ± s.e.m. Data for Sb1800 and Sb1000 in whole brains of individual rats can be found in S6 Data. Statistical maps for group analysis of Sb1800 and Sb1000 can be found in S7 Data. (TIF) [file pbio.2001494.s003.TIF]

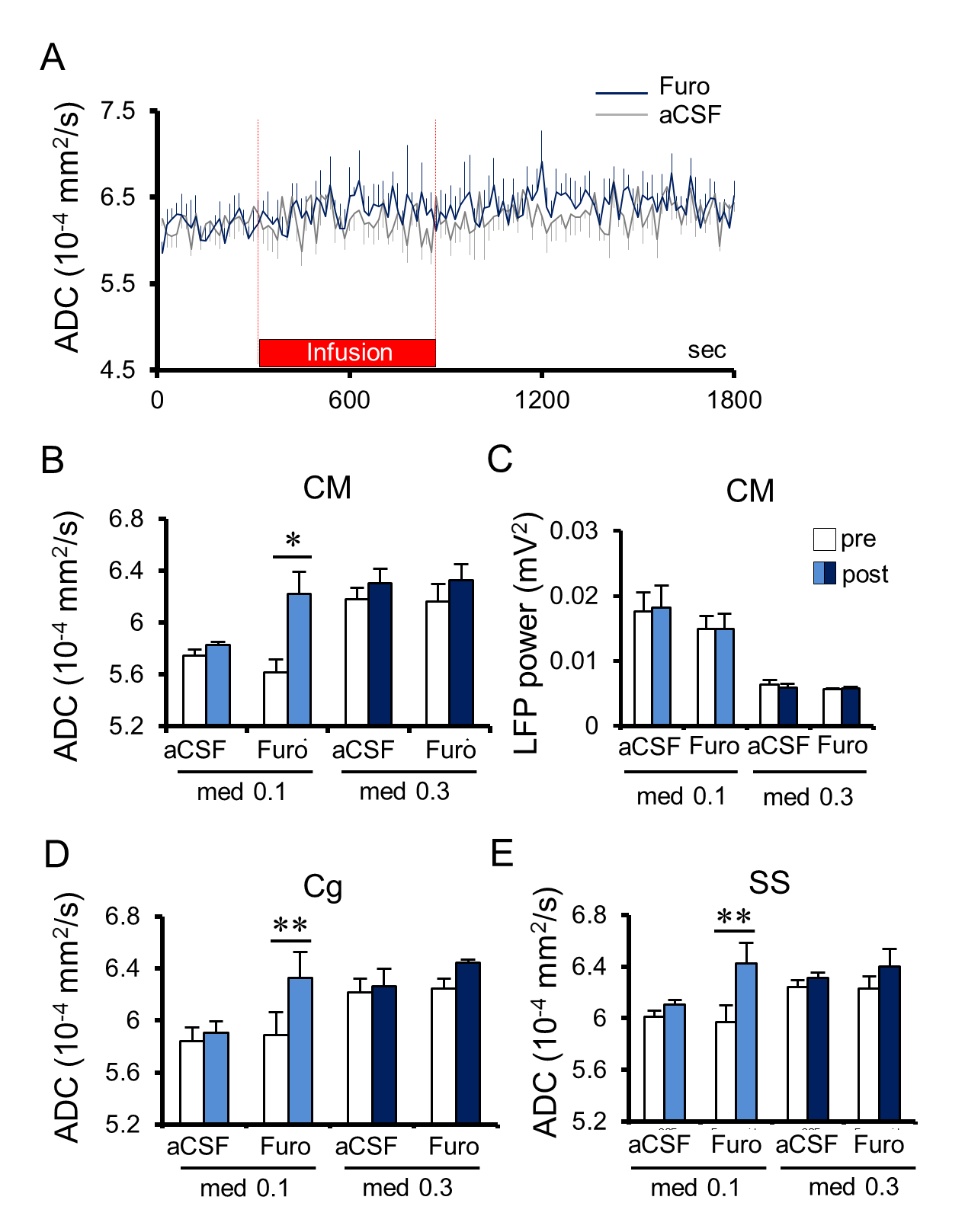

Supplement: S4 Fig — (A) Average time course of ADC change at CM with the infusions of furosemide and aCSF. Average ADC changes in the CM (B), the Cg (D), and the SS (E) pre- and post-infusion of furosemide (n = 6 for each dosage) or aCSF (n = 6 for med 0.1 and n = 5 med 0.3) under 0.1 and 0.3 mg/kg/h medetomidine dosages. (C) Total LFP power (frequency range: 1–70 Hz) at the CM pre- and post-CM infusion with furosemide (n = 6 for each dosage) or aCSF (n = 6 for each dosage) under 0.1 and 0.3 mg/kg/h medetomidine dosages. Time course and bar plots exhibit mean ± s.e.m. * p<0.05, ** p<0.01 (Paired t-test between pre and post). ADC data of individual rats found in S3 Data. LFP power data of individual rats can be found in S4 Data. (TIF) [file pbio.2001494.s004.TIF]

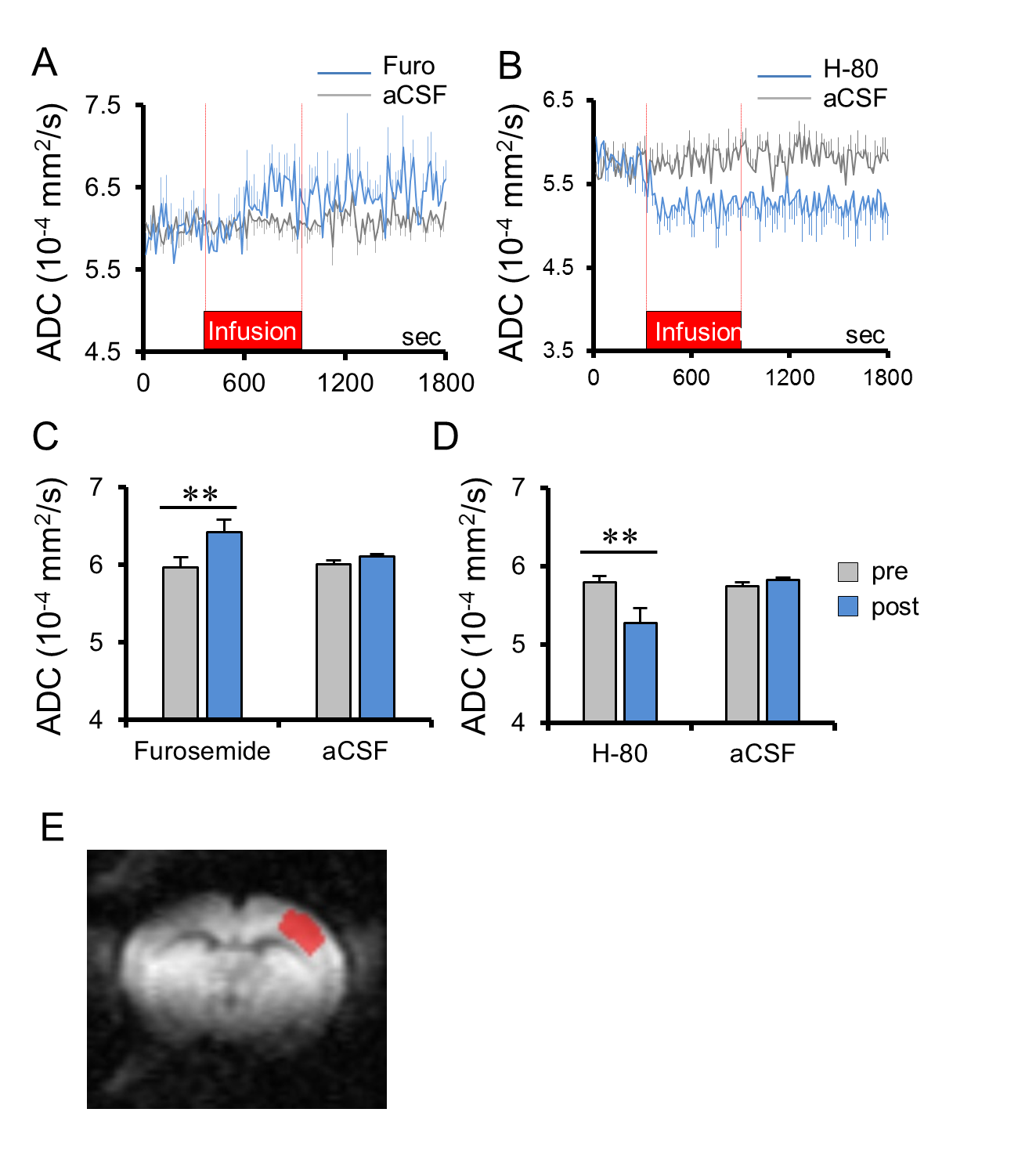

Supplement: S5 Fig — Average time course of ADC change at the SS with CM infusion of aCSF (n = 6), furosemide (A; n = 6), or H-80 (B; n = 6) (SS) under 0.1 mg/kg/h medetomidine condition. The average ADC at the SS pre- and post-CM infusion of aCSF, furosemide (C), or H-80 (D). (E) ROI location (red region) in SS overlaid on the representative DfMRI image of b1000. Time course and bar plots exhibit mean ± s.e.m. ** p<0.01 (Paired t-test between pre and post). ADC data of individual rats found in S3 Data. (TIF) [file pbio.2001494.s005.TIF]
