# Supplementary material for: Water diffusion closely reveals neural activity status in rat brain loci affected by anesthesia
Source: PLoS Biol. 2017 Apr 13;15(4):e2001494. doi: 10.1371/journal.pbio.2001494 (PMC5390968; doi:10.1371/journal.pbio.2001494)
Supplement: S1 Table — Correlation of absolute ADC value or the ratio of BOLD signal change with anesthetic doses at 12 locations and in the whole brain (r: correlation coefficient, p: p value). * p<0.05, ** p<0.01, *** p<0.001. Data for 12 brain locations and whole brain of individual rats can be found in S1 Data for ADC and S2 Data for BOLD. (DOCX) [file pbio.2001494.s006.docx]

**S1 Table**

| Region | ADC | | | |  | BOLD | | | |
| --- | --- | --- | --- | --- | --- | --- | --- | --- | --- |
|  | Iso | | Med | |  | Iso | | Med | |
|  | r | p | r | p |  | r | p | r | p |
| Somatosensory cortex | 0.478 | ** | 0.605 | ** |  | 0.685 | *** | -0.624 | ** |
| Motor cortex | 0.408 | * | 0.073 | - |  | 0.635 | ** | -0.663 | ** |
| Visual cortex | 0.478 | ** | 0.683 | ** |  | 0.512 | * | -0.689 | ** |
| Auditory cortex | 0.424 | ** | 0.499 | * |  | 0.604 | ** | -0.672 | ** |
| Cingulate cortex | 0.495 | ** | 0.111 | - |  | 0.561 | ** | -0.689 | ** |
| Caudate-Putamen | 0.568 | *** | 0.533 | * |  | 0.495 | * | -0.612 | * |
| Amygdala | 0.485 | ** | 0.417 | - |  | 0.516 | * | -0.657 | ** |
| Hippocampus | 0.408 | * | 0.465 | * |  | 0.604 | ** | -0.626 | ** |
| Thalamus | 0.361 | * | 0.463 | * |  | 0.595 | ** | -0.611 | * |
| Hypothalamus | 0.497 | ** | 0.496 | * |  | 0.521 | * | -0.651 | ** |
| Dorsal raphe | 0.363 | * | 0.523 | * |  | 0.514 | * | -0.528 | * |
| Periaqueductal gray | 0.323 | * | 0.365 | - |  | 0.489 | * | -0.531 | * |
| Whole brain | 0.227 | - | 0.214 | - |  | 0.570 | ** | -0.670 | ** |
