# Supplementary material for: Water diffusion closely reveals neural activity status in rat brain loci affected by anesthesia
Source: PLoS Biol. 2017 Apr 13;15(4):e2001494. doi: 10.1371/journal.pbio.2001494 (PMC5390968; doi:10.1371/journal.pbio.2001494)
Supplement: S2 Table — The slope was estimated by dividing the absolute ADC changes or the relative BOLD signal change with anesthetic agent dose changes. * Regions with a significant correlation (p<0.05) as per S1 Table. A.U. = arbitrary units. Data for 12 brain locations and whole brain of individual rats can be found in S1 Data for ADC and S2 Data for BOLD. (DOCX) [file pbio.2001494.s007.docx]

**S2 Table**

| Region | Slope (Absolute ADC change/anesthesia dose unit) | | |  | Slope (Relative BOLD change/anesthesia dose unit) | | |
| --- | --- | --- | --- | --- | --- | --- | --- |
|  | Iso (10^-3^ mm^2^/s/%) |  | Med (10^-3^ mm^2^/s/mg/kg/h) |  | Iso (A.U./%) |  | Med (A.U./mg/kg/h) |
| Somatosensory cortex | 0.141* |  | 0.646* |  | 0.067* |  | -0.364* |
| Motor cortex | 0.140* |  | 0.066 |  | 0.062* |  | -0.351* |
| Visual cortex | 0.154* |  | 0.711* |  | 0.044* |  | -0.362* |
| Auditory cortex | 0.179* |  | 0.910* |  | 0.056* |  | -0.310* |
| Cingulate cortex | 0.147* |  | 0.187 |  | 0.049* |  | -0.316* |
| Caudate-Putamen | 0.200* |  | 0.833* |  | 0.042* |  | -0.264* |
| Amygdala | 0.169* |  | 0.684 |  | 0.050* |  | -0.287* |
| Hippocampus | 0.155* |  | 0.730* |  | 0.057* |  | -0.321* |
| Thalamus | 0.161* |  | 0.743* |  | 0.054* |  | -0.283* |
| Hypothalamus | 0.164* |  | 0.772* |  | 0.049* |  | -0.298* |
| Dorsal raphe | 0.190* |  | 0.986* |  | 0.047* |  | -0.264* |
| Periaqueductal gray | 0.193* |  | 0.745 |  | 0.043* |  | -0.233* |
| Average of 12 brain locations | 0.165 |  | 0.675 |  | 0.052 |  | -0.304 |
| Whole brain | 0.094 |  | 0.218 |  | 0.051* |  | -0.300* |
