# Supplementary material for: Water diffusion closely reveals neural activity status in rat brain loci affected by anesthesia
Source: PLoS Biol. 2017 Apr 13;15(4):e2001494. doi: 10.1371/journal.pbio.2001494 (PMC5390968; doi:10.1371/journal.pbio.2001494)
Supplement: S3 Table — (DOCX) [file pbio.2001494.s008.docx]

**S3 Table**

| Abbreviation | | Name |
| --- | --- | --- |
| Brain regions | SS | Somatosensory cortex |
|  | M | Motor cortex |
|  | V | Visual cortex |
|  | Au | Auditory cortex |
|  | Cg | Cingulate cortex |
|  | CPu | Caudate-Putamen |
|  | Hip | Hippocampus |
|  | Amy | Amygdala |
|  | Tha | Thalamus |
|  | HT | Hypothalamus |
|  | DR | Dorsal raphe |
|  | PAG | Periaqueductal gray |
|  | CM | Central medial thalamic nucleus |
|  | pHT | Posterior hypothalamic nucleus |
|  | vmHT | Ventral medial hypothalamic nucleus |
|  | vlPO | Ventrolateral preoptic nucleus |
|  | VPL | Ventral posterolateral thalamic nucleus |
| Technical terms | aCSF | Artificial cerebrospinal fluid |
|  | ADC | Apparent diffusion coefficient |
|  | BOLD | Blood oxygenation level dependent |
|  | CBF | Cerebral blood flow |
|  | DfMRI | Diffusion functional magnetic resonance imaging |
|  | EPI | Echo planar imaging |
|  | EMG | Electromyography |
|  | FWHM | Full width at half maximum |
|  | H-80 | -80 mOsm hypotonic aCSF |
|  | iso | Isoflurane |
|  | LFP | Local field potential |
|  | MABP | Mean arterial blood pressure |
|  | med | Medetomidine |
|  | RARE | Rapid acquisition with relaxation enhancement |
|  | ROI | Region of interest |
|  | SE | Spin echo |
|  | SNR | Signal:noise ratio |
|  | SPM | Statistical parametric mapping |
